# Psychedelic microdosing benefits and challenges: an empirical codebook

**DOI:** 10.1186/s12954-019-0308-4

**Published:** 2019-07-10

**Authors:** Thomas Anderson, Rotem Petranker, Adam Christopher, Daniel Rosenbaum, Cory Weissman, Le-Anh Dinh-Williams, Katrina Hui, Emma Hapke

**Affiliations:** 1 Department of Psychology, University of Toronto, Mississauga, Canada; 2 Clinical Psychology, York University, Toronto, Canada; 3 Department of Medicine, University of Toronto, Toronto, Canada; 4 Department of Psychiatry, University of Toronto, Toronto, Canada; 5 Department of Psychological Clinical Science, University of Toronto at Scarborough, Toronto, Canada

**Keywords:** Psychedelic, Microdosing, LSD, Psilocybin, Grounded theory, Mood, Depression, Anxiety, Self-efficacy, Open science

## Abstract

**Background:**

Microdosing psychedelics is the practice of consuming very low, sub-hallucinogenic doses of a psychedelic substance, such as lysergic acid diethylamide (LSD) or psilocybin-containing mushrooms. According to media reports, microdosing has grown in popularity, yet the scientific literature contains minimal research on this practice. There has been limited reporting on adverse events associated with microdosing, and the experiences of microdosers in community samples have not been categorized.

**Methods:**

In the present study, we develop a codebook of microdosing benefits and challenges (MDBC) based on the qualitative reports of a real-world sample of 278 microdosers.

**Results:**

We describe novel findings, both in terms of beneficial outcomes, such as improved mood (26.6%) and focus (14.8%), and in terms of challenging outcomes, such as physiological discomfort (18.0%) and increased anxiety (6.7%). We also show parallels between benefits and drawbacks and discuss the implications of these results. We probe for substance-dependent differences, finding that psilocybin-only users report the benefits of microdosing were more important than other users report.

**Conclusions:**

These mixed-methods results help summarize and frame the experiences reported by an active microdosing community as high-potential avenues for future scientific research. The MDBC taxonomy reported here informs future research, leveraging participant reports to distil the highest-potential intervention targets so research funding can be efficiently allocated. Microdosing research complements the full-dose literature as clinical treatments are developed and neuropharmacological mechanisms are sought. This framework aims to inform researchers and clinicians as experimental microdosing research begins in earnest in the years to come.

**Electronic supplementary material:**

The online version of this article (10.1186/s12954-019-0308-4) contains supplementary material, which is available to authorized users.

## Introduction

The practice of microdosing psychedelics involves ingesting sub-hallucinogenic amounts of a psychedelic substance (e.g. LSD, psilocybin) and has recently grown in popularity. The number of popular media accounts and book-length treatments of microdosing has been growing [1–7]. Online microdosing communities have grown to the tens of thousands with more than 40,000 users subscribing to the /r/microdosing subreddit (/r/microdosing subreddit, Reddit Inc, San Francisco, CA, USA). This public interest speaks to a social need for scientific studies to inform the public about the effects of microdosing. Initial scientific investigations of microdosing are just beginning [8–11] (Rosenbaum D, Weissman C, Hapke E, Hui K, Petranker R, Dinh-Williams L-A, et al.: Microdosing psychedelic substances: demographics, psychiatric comorbidities, and comorbid substance use, in preparation) and future directions remain unclear. While full-dose psychedelic research is growing in prominence and outcomes from full-dose studies can certainly inform microdosing studies, focusing solely on known full-dose outcomes could result in missing unanticipated benefits and challenges specific to microdosing. As such, beginning with an open, exploratory approach could result in a better understanding of the potential benefits and challenges specific to microdosing. The present study aims to provide a data-driven taxonomy describing the positive and negative experiences reported by microdosers from an open-ended analysis of microdosing-specific outcomes, summarizing high-potential avenues for focused experimental investigations.

### The benefits of full-dose psychedelics

While more than a thousand early studies linked psychedelic use with beneficial effects [12], there was a 40-year pause on psychedelic research following the prohibition of these substances [13]. Despite continued prohibition, modern research has revealed the promising potential of LSD and psilocybin for treating alcohol and tobacco dependence [14–17], depression [18, 19], and end-of-life anxiety [20–22], while related research on 3,4-methylenedioxymethamphetamine (MDMA) has shown great promise for treating post-traumatic stress disorder [23]. Psychedelics can also increase openness and occasion mystical-type experiences in healthy controls [24–26]. As full-dose psychedelics appear to aide in the relief of severe, chronic psychiatric conditions (e.g. depression, anxiety, PTSD), milder mental health concerns may plausibly be treated by lower, recurring doses. This is especially worth considering if certain full-dose outcomes are found to rely on purely pharmacologic mechanisms rather than primarily on phenomenological experiences [27].

Limiting microdosing research to topics that have been investigated in full-dose research could prematurely overlook unpredicted and potentially distinct microdosing outcomes. Full-dose research has employed various focal assessments of symptomatology, mood, and personality that are likely applicable to microdosing research, but due to the low doses and lack of perceptual alteration intended in microdosing, other full-dose phenomena, such as ego dissolution and mystical-type experiences, are less relevant to microdosing research. Instead, as a means of preparing for a broad range of outcomes, the present work solicited open-ended reports of benefits and challenges. Additionally, as psychedelic substances act on distinct yet overlapping neural receptor sites, it seems plausible that distinct patterns could emerge for different substances. The present study thus included microdosers who used LSD, psilocybin, or both.

### The challenges of full-dose psychedelics

While psychedelics appear to have considerable potential benefits and low physiological risks [28–30], full-dose experiences can put participants under considerable psychological risk [31]. In a survey targeting participants that had at least one challenging experience (“bad trip”) with psilocybin mushrooms, 39% of respondents rated their full-dose experiences as among the top 5 most psychologically difficult/challenging experiences of their lives [32]. Griffiths et al. [20] used both “high” (22 mg/70 kg) and “low” (1 or 3 mg/70 kg) doses of psilocybin as experimental and control conditions, respectively. A dose-response effect could be seen such that in the high-dose condition, 32% of participants reported physiological discomfort whereas only 12% reported the same in the low-dose condition; likewise, 26% reported anxiety in the high-dose condition versus 15% in the low-dose condition [20]. Delayed-onset headaches are another possible side-effect of full-dose psilocybin [33].

To mitigate these risks, Johnson et al. [31] proposed safety guidelines for use with full-dose psychedelic substances, which rely on managing participant inclusion and having a comfortable, guided clinical setting. As microdosing does not involve the intensity of experience present in full-dose research, challenging experiences may be less likely. One may, however, anticipate that less frequent, less intense versions of full-dose challenges could be present even at the very low doses used in microdosing (e.g. restlessness instead of insomnia, mild anxiety instead of fear, mild headaches). As the study of microdosing is in its infancy, we could also expect there to be challenges that fall beyond the scope of reports based on full doses; the present study thus preferred open-ended surveying of drawbacks over pre-existing focal questionnaires.

## Methods

### The present study

In this study, we explored the benefits and challenges experienced by microdosers in a cross-sectional, retrospective, anonymous online survey. Respondents reported their subjective microdosing benefits and challenges (MDBCs) and the subjective importance of each outcome. We then used a grounded theory approach [34] to identify commonly-reported MDBCs and thereby deliver an empirical MDBC taxonomy to support future microdosing research. We also explored whether microdosing substances (LSD-only versus psilocybin-only versus LSD and psilocybin) were associated with different outcomes.

This study was part of a larger project that reported on the demographic and psychiatric comorbidities of the sample (Rosenbaum D, Weissman C, Hapke E, Hui K, Petranker R, Dinh-Williams L-A, et al.: Microdosing psychedelic substances: demographics, psychiatric comorbidities, and comorbid substance use, in preparation) as well as a paper that addressed pre-registered hypotheses concerning mental health, personality, and creativity variables [8].

### Grounded theory method

Microdosers were prompted to provide up to three benefits and up to three challenges associated with microdosing in small on-screen text boxes, resulting in short phrases (e.g. “Amplified emotions and better understanding of them”, “Fear of unknown effects, since its [sic] not studied”) or in one- or two-word responses (e.g. “Creativity”, “Better mood”, “Illegal”, “Too Energetic”). The coding authors (TA and AC) independently coded these benefits and challenges using the principles of classic grounded theory [34–36]. Discrepant codes were periodically discussed until a final set of codes was agreed upon (i.e. saturation was reached). These codes were hierarchically built into three layers of abstraction: codes (level one) were grouped under sub-categories (level two), which were grouped under categories (level three). This hierarchy was iteratively discussed and changes were agreed upon over five refining passes. We incorporated the diction used by the respondents where possible to better reflect the data-driven nature of the final codebook (see Additional file 1 and full online codebook; [37]).

Inter-rater agreement was calculated separately for benefits and challenges and at each level (code, sub-category, category). Agreement was above 85% at every level (benefit code 85.1%, benefit sub-category 89.2%, benefit category 92.6%; challenge code 85.7%, challenge sub-category 86.9%, challenge category 88.5%). Each report was coded twice, once by each coding author, and the sum of coded items in each category was halved; as a result, the frequency of any given category can be a non-integer value (e.g. 807.5 coded benefits, 603.5 coded challenges; “Empirical codebook: benefits of microdosing” and “Empirical codebook: challenges of microdosing” sections).

### Respondents

Participation was voluntary under informed consent, in accord with the Declaration of Helsinki, and was non-remunerative. The sample analysed in the present study includes the 278 respondents that answered the MDBC questions after indicating they had experience with microdosing LSD-only, psilocybin-only, or both LSD and psilocybin; respondents that indicated they used other substances to microdose (e.g. DMT, Salvia divinorum) are not included in the present report, allowing us to focus our efforts on the most commonly reported microdosing substances that are most likely to be studied in future research. Recruitment was primarily via the online forum “Reddit” (Reddit Inc, San Francisco, CA, USA). Reddit is an online forum with self-organizing sub-groups, called “subreddits”, which curate content for their “subscribers”. These subreddits discuss topics of mutual interest, making these communities potential pools of willing participants akin to other crowdsourcing approaches, e.g. Amazon mTurk, CrowdFlower, Prolific [38]. Compared to the US population, Reddit users tend to be younger, educated or seeking a college education, and present in a male-to-female ratio of approximately 2:1 [39] thus this sample’s generalizability is limited to modern Western populations. In the present sample, respondents had a mean age of 27.8 (SD 8.9); age was non-normally distributed with an interquartile range of 21–31 years (median 26.0, range 16–63). Most participants were male (*M* 237, *F* 31, other 10), heterosexual (*N* = 211, other 57), and white or European (*N* = 234, other 44). For a more comprehensive breakdown of all survey respondents, see our epidemiological report, which includes reports on psychiatric disorders (Rosenbaum D, Weissman C, Hapke E, Hui K, Petranker R, Dinh-Williams L-A, et al.: Microdosing psychedelic substances: demographics, psychiatric comorbidities, and comorbid substance use, in preparation). Microdosers from the following subreddits were solicited: Microdosing, Nootropics, Psychonaut, RationalPsychonaut, Tryptonaut, Drugs, LSD, shrooms, DMT, researchchemicals, and SampleSize [40].

### Design and questionnaires

Respondents completed a survey about their microdosing history including microdosing regimen (substance, dose, etc.), subjective benefits and challenges of microdosing, the importance of these benefits and challenges, and focal questions concerning behaviour and consumption changes. For concision, the numerous variables collected but not discussed here are not described here; many are discussed in our previous work [8] (Rosenbaum D, Weissman C, Hapke E, Hui K, Petranker R, Dinh-Williams L-A, et al.: Microdosing psychedelic substances: demographics, psychiatric comorbidities, and comorbid substance use, in preparation) and the complete survey is available online [41]. Here we focus on questions concerning microdosing benefits and challenges (MDBCs), health behaviours, and substance-use changes.

#### Microdosing benefits and challenges (MDBCs)

Microdosing respondents reported up to three benefits and three drawbacks of microdosing psychedelics. They also gave each outcome a rating of subjective importance on a sliding scale from 0 to 100 [42].

#### Improved health behaviours and reduced consumption

Microdosing respondents indicated whether they had, as a result of microdosing, experienced improvements in each of the following domains: mood, anxiety, meditative practice, exercise, eating habits, and sleep. They also indicated whether they had reduced their use of any of the following substances: caffeine, alcohol, cannabis, tobacco, psychiatric prescription medications, and illicit substances. These questions appeared on the page after the open-ended benefits and challenges questions to avoid contamination via priming.

## Results

### Microdosing substances

Respondents reported the substance they used to microdose and were removed if they indicated using substances other than LSD or psilocybin. This sample includes 278 respondents in three categories: LSD-only (*N* = 195), psilocybin-only (*N* = 50), and respondents that have microdosed with LSD and psilocybin (*N* = 33).

### Empirical codebook: benefits of microdosing

Grounded theory analyses resulted in a total of 807.5 coded benefits of microdosing. Taxonomy-building resulted in 46 codes organized into 21 sub-categories and 11 categories. The most frequently reported codes were improved mood (12.8%), improved focus (10.0%), creativity (9.4%), and improved energy (7.6%).

#### Categories of benefit

**Fig. 1:**
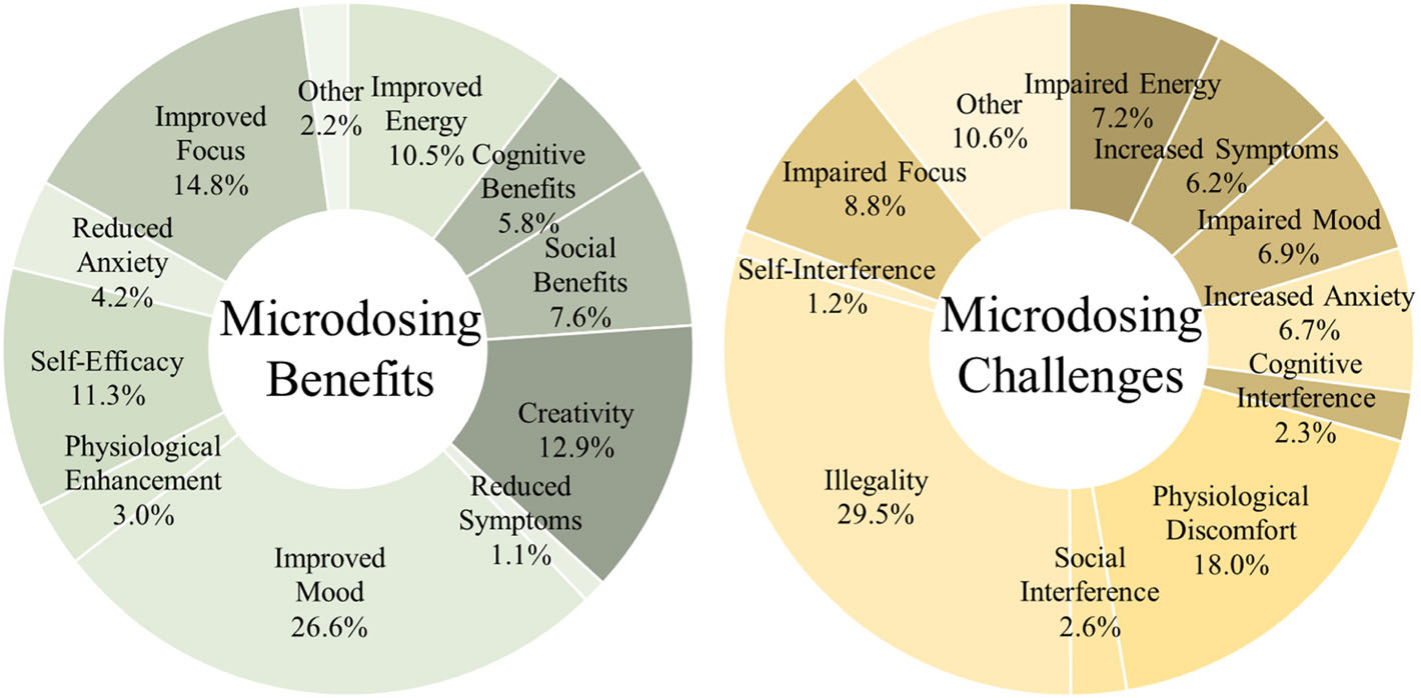
Categories of microdosing benefits and challenges. Values indicate percentage endorsement of outcomes. Values were generated through open-ended responses, and thus magnitude is descriptive and should be used for hypothesis generation. These data indicate reported outcomes, not confirmed effects

This summary provides descriptions of the 11 categories of benefits that were distiled from participant reports (Fig. 1). As per grounded theory, the naming conventions for codes reflect the language used by respondents, but more flexibility was introduced as needed at higher orders of abstraction. Full descriptions of every code are available in the full codebook (see Additional file 1).

Improved mood (26.6%, 215 reports): This most frequently reported benefit-category captures all codes related to mood improvements: happiness, well-being, peace, calm, and reductions in depressive symptoms. Also included are reports of improved outlook, appreciation of life, optimism, spiritual and emotional insights, and being more in touch with emotions.

Improved focus (14.8%, 119.5 reports): This benefit-category references codes concerning focus and concentration, conscious awareness, mindfulness, and increased engagement and attentiveness.

Creativity (12.9%, 104 reports): This category includes creativity per se, as well as meta-creative processes, e.g. shifting perspectives, divergent thinking, curiosity, and openness.

Self-efficacy (11.3%, 91.5 reports): This category references improvements in self-efficacy (motivation/ambition, productivity, confidence, sense of agency) and self-care (introspection, meditation, and other behaviours facilitating mental health).

Improved energy (10.5%, 84.5 reports): This category includes codes referencing “improved energy” per se, as well as alertness, wakefulness, and stimulation.

Social benefits (7.6%, 61 reports): This category references various socially facilitating benefits such as extraversion, empathy, sense of connection, and verbal fluency.

Cognitive benefits (5.8%, 47 reports): This category concerns cognitive enhancement (understanding, problem-solving), clarity of thought (clear headedness, lucidity), and memory.

Reduced anxiety (4.2%, 34 reports): References to anxiety reduction and social-anxiety reduction fit in this category.

Physiological enhancement (3.0%, 24 reports): This category concerns biological processes including enhanced senses (especially visual), cardiovascular endurance, sleep quality, and reduced migraines and/or headaches.

Other perceived benefits (2.2%, 18 reports): This category was a catch-all for otherwise uncategorized codes. These include the novelty of the experience itself, the ability to control the dose, the lack of side-effects, and other miscellany. This category also includes 1 report that there were no beneficial effects.

Reduced symptoms (other) (1.1%, 9 reports): References to stress reduction, reduced sensitivity to trauma, and references to reduced substance dependence (e.g. quitting smoking) are included.

### Empirical codebook: challenges of microdosing

Grounded theory coding resulted in a total of 603.5 coded challenges of microdosing. Taxonomy-building resulted in 44 codes organized into 23 sub-categories and 11 categories. The most frequently reported low-level codes were illegality (10.8%), dose accuracy (9.1%), poor focus (8.8%), and anxiety (5.3%).

#### Categories of challenges

As above, this summary provides extended descriptions of the 11 categories of challenge (Fig. 1).

Illegality (29.5%, 178 reports): This category captures codes concerning the illegality of psychedelic microdosing substances per se, as well as codes concerning the consequences thereof. These include dosing challenges associated with unregulated substances (e.g. taking too much or too little), the availability of the substance (i.e. dealing with the black market), and cost of the substance. Also included is the social stigma surrounding the use of these substances and feeling the need to hide one’s activity from others.

Physiological discomfort (18.0%, 108.5 reports): This category concerns physically detrimental challenges including disrupted senses (visual), temperature dysregulation, numbing/tingling, insomnia, gastrointestinal distress, reduced appetite, and increased migraines and/or headaches.

Impaired focus (8.8%, 53 reports): This challenge category references codes concerning poor focus, distractibility, and absent-mindedness.

Increased anxiety (6.7%, 40.5 reports): References to increased anxiety (general, social, existential) fit in this category.

Impaired energy (7.2%, 43.5 reports): This category includes codes referencing both excessive energy (restlessness, jitters) and inadequate energy (fatigue, drowsiness, brain fog).

Impaired mood (6.9%, 41.5 reports): This category includes codes related to mood deterioration (sadness, discontent, irritability), emotional difficulties (over-emotionality, mood swings), and impaired outlook (fear, feeling unusual).

Social interference (2.6%, 15.5 reports): This category references various socially impairing challenges such as awkwardness, oversharing, and difficulties with sentence-production in social settings.

Cognitive interference (2.3%, 14 reports): This category concerns confusion, disorientation, racing thoughts, and poor memory.

Self-interference (1.2%, 7.5 reports): This category references codes concerning self-processing concerns (dissociation, depersonalization) and self-sabotaging (rumination, over-analysis).

Other perceived challenges (10.6%, 64 reports): This category was a catch-all for otherwise uncategorized codes. These include the unknown risk-effect profile of microdosing itself, the need to prepare and remember to dose, references specifically citing that there were no challenges (1.5%), and other miscellany. This category also includes reports that there were no beneficial effects (0.6%). Furthermore, this category includes substance-related concerns regarding taste, pupil dilation, and duration of effects, and also concerns about negative drug interactions.

Increased symptoms (other) (6.2%, 37.5 reports): References to after effects (psychological dependence and concerns about potential addiction, substance tolerance, comedown or hangover) and also more concerning, but rare, adverse psychological events (0.7%).

### Benefits and challenges by microdosing substance

Subjective importance ratings were non-normally distributed thus Wilcoxon signed rank tests were used to compare between substances. There was a significant difference between the subjective rated importance of benefits based on substance (*W* = 3658, *p <* 0.01, *N*_1_ = 195, *N*_2_ = 50, *d* = 0.353) with psilocybin-only microdosers (median = 87.83, SD = 15.76) rating benefits as significantly more important than LSD-only microdosers (median = 76.67, SD = 14.59); there were no differences found relative to respondents using both LSD and psilocybin (median = 82.33, SD = 14.28, ps > 0.14). The substance-related difference between subjective importance of challenges was non-significant (*W* = 3841.5, *p =* 0.56, *N*_1_ = 177, *N*_2_ = 46, *d* = 0.079) with psilocybin-only microdosers (median = 47.67, SD = 24.98) rating challenges equivalently to LSD-only microdosers (median = 47.5, SD = 24.65); there were no differences found relative to respondents using both LSD and psilocybin (median = 51.67, SD = 23.79, ps > 0.66). Rates at which specific MDBC categories were reported did not differ between LSD-only, psilocybin-only, and LSD and psilocybin respondents (benefits *χ*^2^(20) = 17.26, *p =* 0.636; challenges *χ*^2^(20) = 7.73, *p =* 0.994).

### Improvements and reductions

**Fig. 2:**
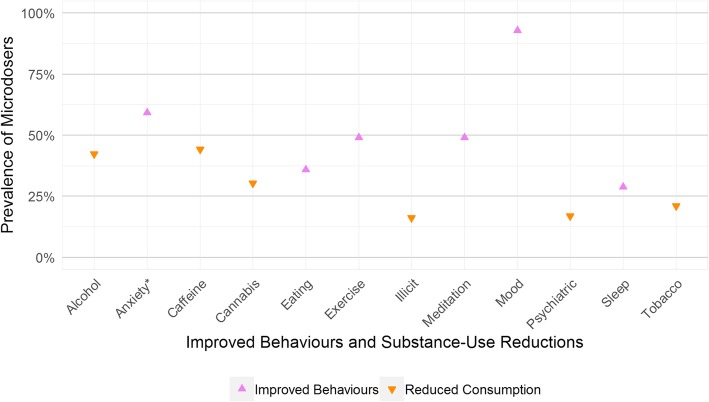
Percentage of microdosers endorsing improved behaviours and reductions in substance-use. Prevalence rate should be used for hypothesis generation as these data indicate reported outcomes, not confirmed effects. *Note: Anxiety refers to improvements to anxiety-related experiences, not to increased experience of anxiety

After reporting open-ended outcomes, participants answered targeted questions concerning behavioural improvements and substance-use reductions (Fig. 2). Respondents reported improved mood (92.9%), anxiety (59.2%), meditative practice (49.1%), exercise (49.1%), eating habits (36.0%), and sleep (28.8%). They also indicated reduced use of caffeine (44.2%), alcohol (42.3%), cannabis (30.3%), tobacco (21.0%), psychiatric prescription medications (16.9%), and illicit substances (16.1%).

## Discussion

Surveying extant communities of microdosers allowed for the creation of an initial qualitative taxonomy of MDBCs. These empirically-grounded MDBCs can inform future microdosing research by leveraging participant reports for high-potential intervention targets so research time and funding can be efficiently allocated. For example, microdosers often report changes in mood, focus, and creativity thus these constructs should be targeted in future intervention research. Concerns of physiological discomfort and restlessness were also commonly reported thus they should also be monitored.

While the improvements and reductions reported by respondents sound promising, they cannot be disentangled from expectation and placebo effects or recall biasses. Furthermore, the MDBC findings cannot indicate causation as this study was observational, not experimental. With these caveats in mind, we discuss how researchers can use these initial findings in their future studies. While necessarily inconclusive due to their exploratory nature, these results point to potential therapeutic effects warranting future placebo-controlled microdosing research.

### Emergent parallelism

Major parallels between benefits and challenges emerged among outcomes. Specifically, each category of outcome is seen as both a benefit and a challenge, other than creativity and illegality (Table 1). This kind of mirroring suggests two hypotheses concerning microdosing: (1) placebo effects and expectancy play a major role in reported effects and/or (2) individual differences moderate reported effects.

**Table 1:** Parallels between benefits and challenges

Outcome category	Benefit category	Challenge category
Mood	Improved mood	Impaired mood
Self	Self-efficacy	Self-interference
Focus	Improved focus	Impaired focus
Social	Social benefits	Social interference
Energy	Improved energy	Impaired energy
Cognitive	Cognitive benefits	Cognitive interference
Anxiety	Reduced anxiety	Increased anxiety
Physiological	Physiological enhancement	Physiological discomfort
Symptoms	Reduced symptoms (other)	Increased symptoms (other)
Other	Other perceived benefits	Other perceived challenges

The first and most parsimonious hypothesis that could explain the parallelism between benefits and challenges is that the effects cancel out and nothing replicable is happening. The presence of opposite outcomes with a net-zero effect is what might be expected in an inactive condition dominated by noise. For example, if microdosing has no effect, random variation might result in some participants reporting decreased anxiety while others report increased anxiety. It may also be the case that microdosing interacts with expectancy in some way, enhancing the effect of expectancy and thus the outcomes could differ even more than anticipated based on the mind-set of the microdoser. Indeed, “set and setting” are major components of full-dose psychedelic use and expectancy is understood to greatly alter the outcome potentials of full-dose psychedelics [31]. Perhaps “set and setting” are also of importance in microdosing, though this remains to be tested. Indeed, each of the constructs described in this taxonomy should be directly tested in placebo-controlled trials.

Nevertheless, there are plausible pharmacological mechanisms of action for microdosing, and it is possible that individual differences in genetically mediated substance metabolism, psychopathological diagnoses and personality, and momentary interpretations of interoceptive signals affect how microdosing outcomes manifest. The HTR2A gene, which encodes the serotonin 5HT-2A receptor, can have various mutations [43] which, alongside other genetic and epigenetic influences, play a role in how 5HT-2A agonists, including LSD and psilocybin, are processed neuropharmacologically. As such, individual differences in receptor sensitivity may moderate optimal microdosing doses, substance choice, and dosing schedule. Genetic and epigenetic factors also influence psychopathology and personality, which can moderate responses to psychedelics [44]. For example, a person with a mood disorder (e.g. major depression) may find that microdosing has a different effect than a person scoring in the healthy range on a depression inventory. One possibility is that increasing between-network functional connectivity could disrupt the patterned use of cortical networks overly favoured under a specific pathology (e.g. to disrupt the greater functional connectivity between the DMN and subgenual prefrontal cortex seen in depression; [45]). In contrast, altering the functional connectivity in a healthy brain could plausibly produce undesirable activity rather than maintain healthy network coherence [46, 47]. Indeed, even in non-pathological participants, top-down interpretations of interoceptive events could cast physiological experiences (e.g. arousal) in a negative light (e.g. restlessness) rather than a positive one (e.g. wakefulness). These different interpretations may be amenable to intervention by preparing participants for certain physiological outcomes [31] whereas the genetic, epigenetic, and psychopathological features could constitute more stable predictors. These moderation hypotheses remain for future research.

**Fig. 3:**
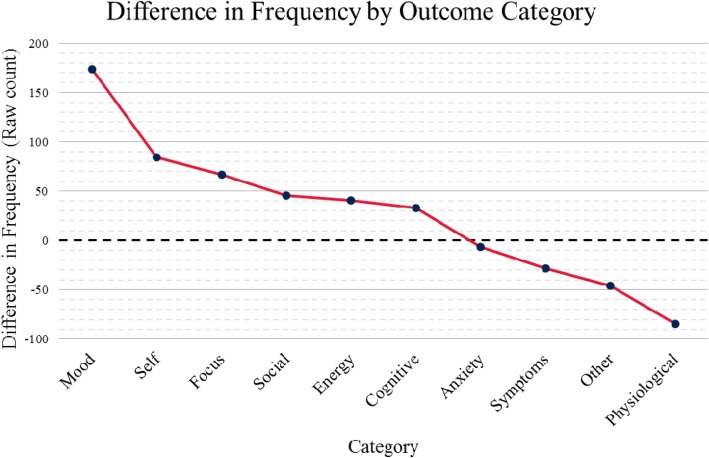
Difference in raw count of reported benefits and challenges. Positive values indicate greater endorsement of benefits in the indicated category; negative values reflect greater endorsement of challenges. Comparisons are exploratory thus differences, regardless of magnitude, should be used for hypothesis generation. These data indicate perceived outcomes and do not indicate confirmed effects

While parallelism emerged, not all categories were equally reported on both sides of the benefit/challenge divide (Fig. 3). When calculating the difference between how often categories of benefits were reported versus how often the parallel challenge-category was reported, the three largest differences in raw reporting rates were mood being more often improved (215 as benefit versus 41.5 as challenge), self-efficacy being more often increased (91.5 benefit, 7.5 challenge), and physiological response being more often discomforting (24 benefit, 108.5 challenge). These categories may provide especially promising starting points for future microdosing research. Anxiety was closest to even with the difference being only 6.5 reports (34 benefit, 40.5 challenge).

### Unique outcomes

Parallelism between benefits and challenges was not universal. The taxonomy includes both unique beneficial and detrimental outcomes: (1) creativity and (2) illegality.

Creativity was the third most common benefit category, and there was no opposite challenge (i.e. participants did not report that microdosing made them less creative or more closed-minded). Microdosers report enhanced creativity and meta-creative processes, such as perspective-shifting/divergent thinking and openness/curiosity. These findings accord with other findings that microdosers have higher creativity than non-microdosers [8, 11] and with full-dose research showing increased openness after full-dose psilocybin [24]. Early psychedelic research preliminarily investigated creativity enhancement and problem-solving [48], and this exciting topic could again be subject to study. Future studies should initially measure various aspects of creativity—e.g. divergent thinking, convergent thinking, insight [8, 11, 49, 50]—to inform more focal investigations on how microdosing may affect creativity.

Illegality was the most commonly reported microdosing challenge. It is notable that the most frequently reported “outcome” is a socio-cultural circumstance, not an outcome of microdosing per se. Psychedelics were made illegal by the UN Convention on Psychotropic Substances in 1971 and remain so today [13, 51]. Illegality has resulted in a thriving black market economy for illicit substances, both in-person and online [52]. This unregulated criminal market results in unpredictable substance purity, dose accuracy, supply availability, and cost. Illegality has further societal consequences, namely the social stigma associated with substance use, even though psychedelic substances have a relatively benign safety profile compared to other substances, including several legal substances [53]. As such, researchers have begun calling for the legal rescheduling of psychedelic substances [54].

### Improvements and reductions

In addition to the emergent qualitative categories, participants reported on several a priori focal outcomes (Fig. 2). Nine-tenths of respondents endorsed that microdosing improved their mood, which is in agreement with improved mood being the most commonly reported benefit-category. Anxiety improvement was also notable with 59% of respondents indicating this benefit. These rates of reported improvement suggest future research into microdosing for mood and anxiety may be warranted, complementing the recent work treating depression and anxiety with psilocybin [19, 20].

Participants also indicated decreased use of caffeine, alcohol, cannabis, and tobacco (Fig. 2). These findings align with research on full-dose psychedelics: LSD and psilocybin may promote reduced alcohol abuse [14, 16], and psilocybin can have potent long-term reductions in smoking [55]. Microdosing could be investigated as a potential complement, supplement, or alternative to full-dose interventions for smoking cessation or substance use disorders.

## Limitations and future directions

The intent of the present study was to inform empirically-grounded data-collection initiatives by providing high-potential outcomes deserving of further study, while also showcasing challenges that warrant measurement and suitable caution. The intent of the present study was not to make causal claims. We employed no experimental manipulation or longitudinal component, could not control for substance purity, schedule, or dose, nor for prior experience with full-dose psychedelics, and we cannot account for recall bias or placebo effects. MDBCs described here reflect the reports of microdosers, but we cannot claim that these perceived outcomes are causally related to microdosing. LSD and psilocybin were the most frequently used substances and, as microdosing continues to be culturally, scientifically, and clinically relevant, it will be important to establish dose-dependent outcomes of microdosing and to consider the different contexts in which micro- and full doses may be variably appropriate, including when they may complement each other.

Our participant recruitment strategy relied on self-selection and sampled primarily from Reddit; this strategy may have introduced demographic biasses, and these data should not be considered epidemiologically definitive (see Rosenbaum et al. (Rosenbaum D, Weissman C, Hapke E, Hui K, Petranker R, Dinh-Williams L-A, et al.: Microdosing psychedelic substances: demographics, psychiatric comorbidities, and comorbid substance use, in preparation) for further discussion). More than 70% of the sample reported countries of Anglo-cultural origin, and this sample is limited in the sense that it does not reflect a random sampling of the human population. We sought a sample of psychedelic microdosers, a group that may not be randomly distributed in the population, thus this convenience sample is still informative. Nevertheless, future intervention work should endeavour to recruit more inclusive and representative samples.

Qualitative research is, by its nature, biassed by the research team and their coding decisions. MDBCs were processed by two interdependent coders (TA and AC) that iteratively constructed the agreed-upon codebook. Hypothesis-driven coding was avoided to maintain code-integrity [36] and, supporting transparency and re-analysis, both the coded and raw data have been made available [41]. Another taxonomy could emerge from different investigators pursuing more targeted research questions, so these MDBCs should not be taken as definitive. The present taxonomy offers a foundation from which future focal research can be built.

Ultimately, pre-registered randomized placebo-controlled trials (RCTs) of microdosing psychedelics are needed to test its safety and efficacy. Using the MDBC taxonomy as a starting point, appropriate measures can be included to investigate the causal outcomes of microdosing and the mechanisms underlying those outcomes. The potential of microdosing is not yet well understood, but the benefits reported in this taxonomy suggest potential novel research avenues for psychedelic-based pharmacotherapeutic treatment of depression, anxiety, ADHD, smoking cessation, and substance use disorders. Exploring the potential of microdosing for creativity is also warranted.

## Conclusion

Here we provide an initial taxonomy of benefits and challenges associated with psychedelic microdosing, which compliments the other reports built from this larger microdosing research project [8] (Rosenbaum D, Weissman C, Hapke E, Hui K, Petranker R, Dinh-Williams L-A, et al.: Microdosing psychedelic substances: demographics, psychiatric comorbidities, and comorbid substance use, in preparation). The findings presented here suggest a number of potential microdosing research avenues, though experimental, hypothesis-driven studies are needed. The MDBC taxonomy, behavioural improvements, and substance-use reductions warrant RCTs to test therapeutic safety and efficacy of microdosing psychedelics. Online microdosing communities have grown to the tens of thousands, speaking to a social need for scientific study to inform the public about the effects of microdosing. Microdosing research could help inform future psychedelic research by investigating the potential for mixing or contrasting micro- and full-dose psychedelic psychotherapies. We call researchers to do this work following the principles of open science and share our resources accordingly [41]. After a 40-year moratorium, the psychedelic renaissance has begun: rigorous scientific methods can now be used to investigate psychedelics as potential medicines and for “the betterment of well people” [1].

## Additional file

Additional file 1: The complete code hierarchy is available in Additional file 1. (PDF 89 kb)

## Data Availability

The dataset supporting the conclusions of this article is available on the Open Science Framework (osf.io/g5cwy/).
