# Supplementary material for: Psychedelic microdosing benefits and challenges: an empirical codebook
Source: Harm Reduct J. 2019 Jul 10;16:43. doi: 10.1186/s12954-019-0308-4 (PMC6617883; doi:10.1186/s12954-019-0308-4)
Supplement: Supplementary file 1 — The complete code hierarchy is available in Additional file 1. (PDF 89 kb) [file 12954_2019_308_MOESM1_ESM.pdf]

Microdosing Benefits

Categories: 11 ***Concepts: 21*** Codes: 46

Creativity	Improved Mood	Improved Focus	Self-Efficacy	Improved Energy	Social Benefits	Cognitive Benefits	Reduced Anxiety	Physiological Enhancement	Other Perceived Benefits	Reduced Symptoms (Other)
Concepts: 2	***Concepts: 3***	***Concepts: 2***	***Concepts: 2***	***Concepts: 1***	***Concepts: 1***	***Concepts: 1***	***Concepts: 1***	***Concepts: 3***	***Concepts: 3***	***Concepts: 2***
Codes: 3	Codes: 7	Codes: 4	Codes: 7	Codes: 4	Codes: 4	Codes: 3	Codes: 2	Codes: 4	Codes: 5	Codes: 3
Creativity<br>Creativity<br>***Meta-Creative Processes***<br>Perspective Shifting<br>Openness	***Improved Mood***<br>Improved Mood<br>Reduced Depression<br>Calm<br>***Emotional Intelligence***<br>Emotional Intelligence<br>***Improved Outlook***<br>Spirituality<br>Appreciation Positive Outlook	***Improved Focus***<br>Improved Focus<br>***Awareness***<br>Heightened Awareness<br>Mindful Presence<br>Engagement	***Self-Efficacy***<br>Improved Motivation<br>Improved Productivity<br>Confidence<br>Sense of Agency<br>***Self-Care***<br>Self-Care<br>Introspection<br>Improved Meditation	***Improved Energy***<br>Improved Energy<br>Alertness<br>Wakefulness<br>Stimulation	***Social Facilitation***<br>Sociability<br>Empathy<br>Connection<br>Improved Communication Skills	***Cognitive Enhancement***<br>Cognitive Enhancement<br>Clarity of Thought<br>Memory	***Reduced Anxiety***<br>Reduced Anxiety<br>Reduced Social Anxiety	***Physiological Enhancement***<br>Physiological Enhancement<br>Sleep<br>***Reduced Aches***<br>Reduced Headaches<br>***Sensory Enhancement***<br>Improved Senses	***Novelty***<br>Novelty<br>***Misc***<br>Lack of Side-Effects<br>Dose Control<br>Misc<br>***None***<br>None	***Reduced Stress***<br>Reduced Stress<br>Reduced Trauma Sensitivity<br>***Reduced Substance Dependence***<br>Reduced Substance Dependence

Microdosing Challenges

Categories: 11 ***Concepts: 23*** Codes: 44

**Illegality**<br>***Concepts: 3***<br>Codes: 5	**Impaired Mood**<br>***Concepts: 3***<br>Codes: 7	**Impaired Focus**<br>***Concepts: 1***<br>Codes: 1	**Self-Interference**<br>***Concepts: 2***<br>Codes: 2	**Impaired Energy**<br>***Concepts: 2***<br>Codes: 2	**Social Interference**<br>***Concepts: 1***<br>Codes: 2	**Cognitive Interference**<br>***Concepts: 1***<br>Codes: 3	**Increased Anxiety**<br>***Concepts: 1***<br>Codes: 3	**Physiological Interference**<br>***Concepts: 3***<br>Codes: 6	**Other Perceived Challenges**<br>***Concepts: 4***<br>Codes: 9	**Increased Symptoms (Other)**<br>***Concepts: 2***<br>Codes: 4
Illegality<br>Illegality<br>***Substance Reliability***<br>Dose Accuracy<br>Substance Availability<br>Financial Cost<br>***Stigma***<br>Stigma	***Impaired Mood***<br>Negative Emotionality<br>Irritability<br>***Emotional Difficulty***<br>Emotional Depth<br>Emotional Instability<br>Mania<br>***Impaired Outlook***<br>Unusualness<br>Fear	***Impaired Focus***<br>Poor Focus	***Self-Interference***<br>Dissociation<br>***Self-Sabotage***<br>Rumination	***Excess Energy***<br>Restlessness<br>***Inadequate Energy***<br>Fatigue	***Social Difficulty***<br>Social Overengagement<br>Reduced Communication Skills	***Cognitive Interference***<br>Confusion<br>Racing Thoughts<br>Memory	***Increased Anxiety***<br>Anxiety<br>Social Anxiety<br>Existential Anxiety	***Physiological Interference***<br>Sleep Disturbances<br>GI Issues<br>Decreased Appetite<br>***Increased Aches***<br>Body Discomfort<br>Headache<br>***Sensory Disruption***<br>Visual Effects	***Unknown Risk-Effect Profile***<br>Unknown Risk-Effect Profile<br>***Misc***<br>Misc<br>Preparation<br>Lack of Positive-Effects<br>***Substance Effects***<br>Negative Drug Interactions<br>Pupil Dilation<br>Bad Taste<br>Duration Concerns<br>***None***<br>None	***After Effects***<br>Psychological Dependence<br>Tolerance<br>Comedown<br>***Adverse Psychological Events***<br>Adverse Psychological Events
